# Identification of a novel nonsense mutation in *RP1* that causes autosomal recessive retinitis pigmentosa in an Indonesian family

**Published:** 2012-10-03

**Authors:** Anna M. Siemiatkowska, Galuh D.N. Astuti, Kentar Arimadyo, Anneke I. den Hollander, Sultana M.H. Faradz, Frans P.M. Cremers, Rob W.J. Collin

**Affiliations:** 1 Department of Human Genetics, Radboud University Nijmegen Medical Centre, Nijmegen, The Netherlands; 2 Department of Ophthalmology, Radboud University Nijmegen Medical Centre, Nijmegen, The Netherlands; 3 Nijmegen Centre for Molecular Life Sciences, Radboud University Nijmegen Medical Centre, Nijmegen, The Netherlands; 4 Division of Human Genetics, Center for Biomedical Research, Faculty of Medicine, Diponegoro University, Semarang, Indonesia; 5 Department of Ophthalmology, Faculty of Medicine, Diponegoro University/Dr. Kariadi Hospital, Semarang, Indonesia

## Abstract

**Purpose:**

The purpose of this study was to identify the underlying molecular genetic defect in an Indonesian family with three affected individuals who had received a diagnosis of retinitis pigmentosa (RP).

**Methods:**

Clinical evaluation of the family members included measuring visual acuity and fundoscopy, and assessing visual field and color vision. Genomic DNA of the three affected individuals was analyzed with Illumina 700k single nucleotide polymorphism (SNP) arrays, and homozygous regions were identified using PLINK software. Mutation analysis was performed with sequence analysis of the retinitis pigmentosa 1 (*RP1*) gene that resided in one of the homozygous regions. The frequency of the identified mutation in the Indonesian population was determined with TaqI restriction fragment length polymorphism analysis.

**Results:**

A novel homozygous nonsense mutation in exon 4 of the *RP1* gene, c.1012C>T (p.R338*), was identified in the proband and her two affected sisters. Unaffected family members either carried two wild-type alleles or were heterozygous carriers of the mutation. The mutation was not present in 184 Indonesian control samples.

**Conclusions:**

Most of the previously reported *RP1* mutations are inherited in an autosomal dominant mode, and appear to cluster in exon 4. Here, we identified a novel homozygous p.R338* mutation in exon 4 of *RP1*, and speculate on the mutational mechanisms of different *RP1* mutations underlying dominant and recessive RP.

## Introduction

Retinitis pigmentosa (RP, OMIM 268000) represents a clinically heterogeneous group of progressive inherited retinal disorders that primarily affect rod photoreceptor cells, followed by secondary cone photoreceptor cell degeneration [1-3]. RP is the most frequent cause of inherited blindness, affecting approximately 1 in 3,500 to 1 in 5,000 people worldwide. This disease is initially characterized by night blindness followed by visual field constriction that can ultimately lead to legal blindness at a later stage [4].

In addition to the clinical diversity of RP, the disease is genetically heterogeneous. RP can be inherited in an autosomal dominant (adRP, 30% to 40%), autosomal recessive (arRP, 50% to 60%, with sporadic cases accounting for about 45% of all RP [4]), or X-linked (xlRP, 5% to 20%) [1,4], and rarely in a digenic manner [3,5]. At present, of 55 genes known to be mutated in individuals with RP, most display allelic heterogeneity, and some have been implicated in both dominant and recessive modes of inheritance e.g., bestrophin 1 (*BEST1*), neural retina leucine zipper (*NRL*), nuclear receptor subfamily 2, group E, member 3 (*NR2E3*), rhodopsin (*RHO*), retinitis pigmentosa 1 (*RP1*), and retinal pigment epithelium-specific protein 65 kDa (*RPE65*); RetNet-Retinal Information Network; provided in the public domain by the University of Texas Houston Health Science Center, Houston, TX).

Mutations in the retinitis pigmentosa 1 gene (*RP1*, OMIM 603937) are thought to account for approximately 5.5% of adRP cases and only 1% of arRP cases, depending on the type and the position of the mutations [6]. To date, more than 50 disease-causing mutations in the *RP1* gene have been identified. They are predominantly frameshift or nonsense mutations in individuals with dominant RP, clustered in a region spanning codons 500–1053 in exon 4 [6-24]. Only a few cases of recessive *RP1* mutations in exon 4 have been reported [6,20,25-29], the majority of which is located very close to the 3′ end of exon 4. Here, we report an early truncating mutation in the 5′ part of exon 4 (c.1012C>T; p.R338*), which we found was homozygously present in an Indonesian family segregating arRP. A heterozygous carrier of this nonsense mutation did not display any clinical abnormalities characteristic of RP. Therefore, our findings suggest that this particular null allele of *RP1* causes recessive rather than dominant RP.

## Methods

### Subjects

**Figure 1:**
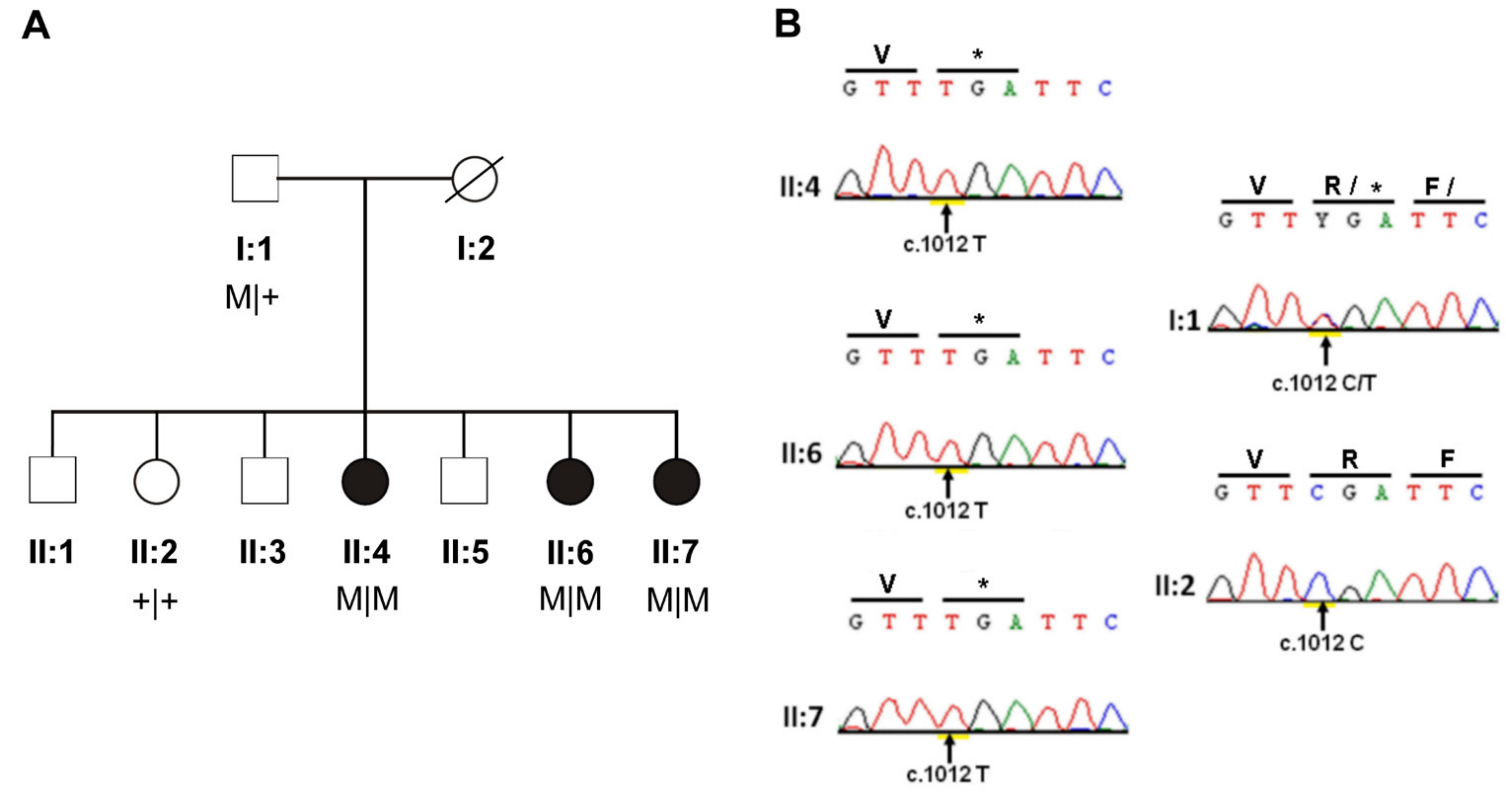
Pedigree structure and mutation analysis of the Indonesian arRP family. **A**: In the family pedigree, affected individuals are indicated with filled symbols whereas unaffected relatives are indicated with open symbols. Genotypes are indicated below the pedigree symbols. M represents the c.1012C>T: (p.R338*) substitution, and + represents the wild-type allele. **B**: Shown are sequence chromatograms of *RP1* corresponding to the three affected individuals (II:4, II:6, II:7), the proband’s father I:1), and one unaffected sibling (II:2). The substituted nucleotides are indicated with arrows.

An Indonesian family of Javanese origin with three affected individuals and two available unaffected relatives was included in this study (Figure 1). Affected family members were diagnosed by an ophthalmologist at the Dr. Kariadi Hospital in Semarang, Central Java, Indonesia. This study was approved by the ethical review boards of the centers involved. Informed consent adhering to the tenets of the Declaration of Helsinki was obtained from all participating affected individuals and unaffected family members. In addition, 184 ethnically matched and unrelated control individuals took part in this study.

### Clinical characterization

The three affected individuals (II:4, II:6, and II:7) and two unaffected relatives (I:1 and II:2) underwent a detailed ophthalmologic examination that included evaluation of visual acuity measurement, fundoscopy, and fundus photography after pupillary dilation. Color vision was tested using Ishihara plates. The size and extent of the visual field defects were assessed with a Humphrey Visual Field Analyzer (Carl Zeiss Meditec, Dublin, CA). Fundus photographs were taken using a Visucam Pro NM (Carl Zeiss Meditec).

### Homozygosity mapping

EDTA blood samples were obtained from all participants. Total genomic DNA was extracted from peripheral leukocytes using a standard salting out procedure [30]. DNA aliquots of each individual were stored at −20 °C. Affected individuals were genotyped on Infinium Human OmniExpress 700 K arrays (Illumina, San Diego, CA) containing approximately 700,000 single nucleotide polymorphisms (SNPs). Array experiments were performed according to the protocol provided by the manufacturer. Homozygous regions were determined using PLINK software [31], with parameters manually set at a cut-off of 1 Mb, and allowing two heterozygous SNPs and ten missing SNPs per window of 50 SNPs.

Homozygous regions were ranked based on the size of the regions. The SNP positions were derived from the UCSC Human Genome Browser build NCBI36/hg18, March 2006.

### Mutation analysis

Primers for *RP1* were designed using Primer3 software [32] and are listed in Table 1. All coding exons and exon-intron boundaries of the *RP1* gene (NM_006269) were amplified with touch-down polymerase chain reaction (PCR) from 50 ng of genomic DNA. PCR reactions were performed in a volume of 25 µl containing 50 ng genomic DNA, 0.2 µM of each primer, 2 mM MgCl_2_, 1 mM deoxyribonucleotide triphosphates, PCR buffer provided by the manufacturer, and 0.5 U Taq polymerase (cat. no. 18,038–042; Invitrogen, Breda, The Netherlands).

**Table 1:** Primer sequences for the amplification of the *RP1* gene.

**Primer**	**Sequence (5′-3′)**	**Product size (base pairs)**
RP1 exon1 F	CCATGTATTCGCTATGGTGC	421
RP1 exon1 R	TGTCCAGGTCTACAGGCTGC	
RP1 exon2 F	GGCAGGCACAGCATCAC	434
RP1 exon2 R	CACCATTCATATCCCACACG	
RP1 exon3 F	TTCAAGCCTAGGAGGTTGTTG	357
RP1 exon3 R	ATTGAAGCATGGATTTTGCC	
RP1 exon4_1 F	GATATTTCTAACTTCTCTGCCTTCC	595
RP1 exon4_1 R	CCCTGGATGATATCTGTGTCC	
RP1 exon4_2 F	ATCAAGAGGGCAGTTTGGC	592
RP1 exon4_2 R	TTGAAGTTCTTGATACCAGTTTTG	
RP1 exon4_3 F	TCACATAATAATGGTTTGCCATC	599
RP1 exon4_3 R	TTTCTATGGAAATTCTTGGAAATC	
RP1 exon4_4 F	TCCCCTTAAAGGAGGGATAC	580
RP1 exon4_4 R	AATTGAATGATGAGCAATAGCC	
RP1 exon4_5 F	GAATGGCAAAGAAGAGTTTAGTTTC	739
RP1 exon4_5 R	ACTGAAGCTTGCAATTGGTG	
RP1 exon4_6 F	GCTTATTTGGTTCCCCTGC	678
RP1 exon4_6 R	AGAGCAACCTCCATCCAAAG	
RP1 exon4_7 F	ACTTGAAAGCTGCTGTTGCC	659
RP1 exon4_7 R	GCTTAAATTACTGACATTTTGATGTG	
RP1 exon4_8 F	CAATGTCTGCAATACCATTGAC	658
RP1 exon4_8 R	TCCTTCATTGGTCTCCTTTTC	
RP1 exon4_9 F	TTAATCCAAGAAGAGGTAGAGGC	616
RP1 exon4_9 R	CCTGGAATTCCTGCAACATAG	
RP1 exon4_10 F	TGGAATTTCAGTGTTCCAGG	592
RP1 exon4_10 R	TGATGACTACCCTTCTCCTCTG	
RP1 exon4_11 F	CATGGTAGTGACTCAGAACCTTTTC	590
RP1 exon4_11 R	CCTTCTTCCTCTAACCCCAAG	
RP1 exon4_12 F	GATAATGCCATTGGTGATATATTTG	600
RP1 exon4_12 R	CGTATTCGTCACATGTGCTTC	

PCR products were purified on NucleoSpin Plasmid QuickPure columns (Macherey-Nagel GmbH & Co, Duren, Germany) and analyzed either in the sense or antisense direction using dye termination chemistry (BigDye Terminator Cycle Sequencing Kit version 3.1; on a 3130 DNA automated sequencer; Applied Biosystems, Foster City, CA). Segregation analysis of the *RP1* mutation identified in the family was performed with sequence analysis. The frequency of this mutation in 184 unrelated and unaffected ethnically matched Indonesian controls was assessed by digesting PCR products with TaqI (New England Biolabs, Ipswich, MA), the recognition site of which is abolished by the alteration in *RP1*.

## Results and Discussion

**Figure 2:**
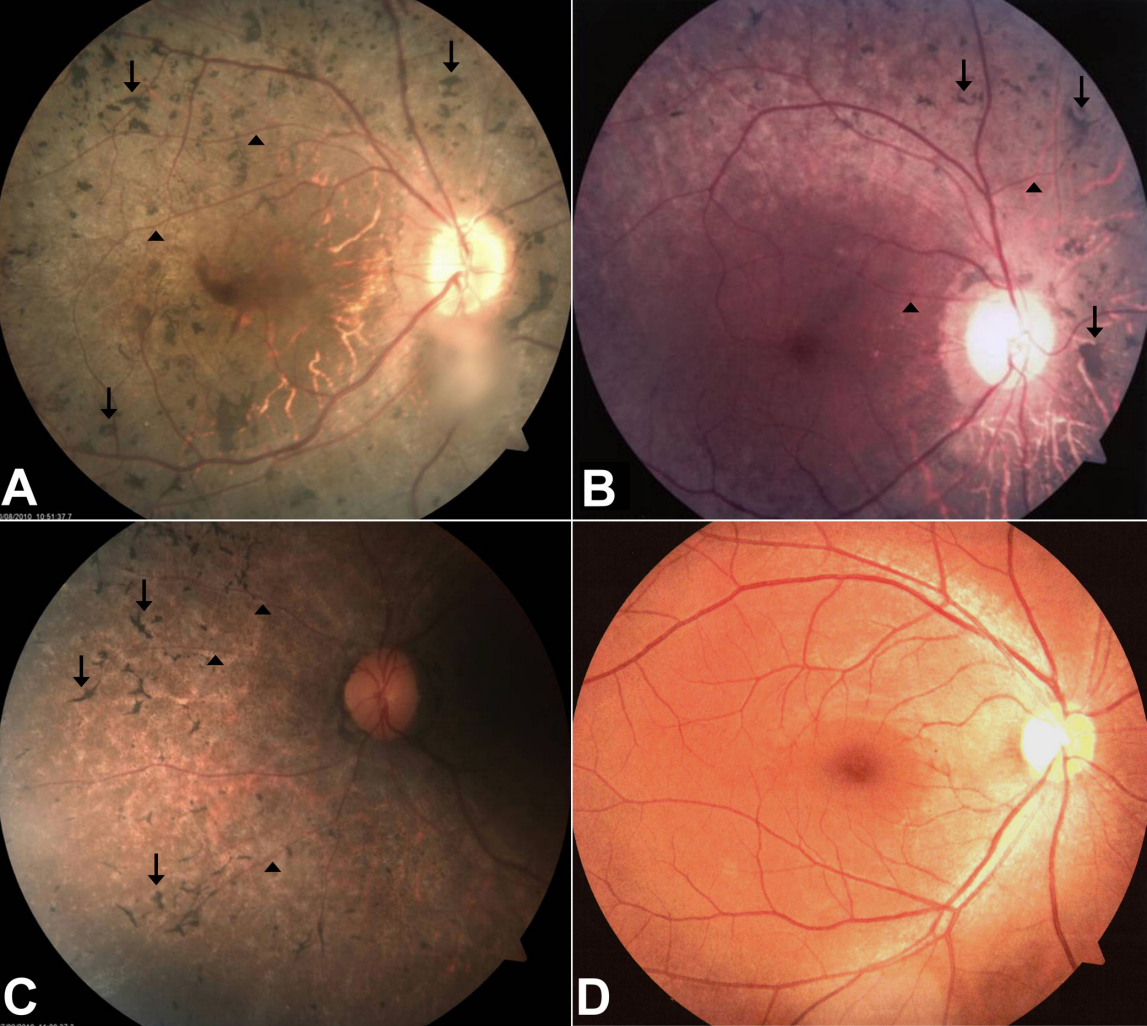
Fundus photographs of affected individuals. Fundus photographs of the right eye from affected individual II:4 **A**: affected individual II:6 **B**: affected individual II:7 **C**: and the unaffected father I:1 **D**: who carries the mutation heterozygously. The ages at the time of investigation of individuals II:4, II:6, and II:7 were 36, 28, and 26, respectively. Fundus examination of the affected individuals revealed typical features of retinitis pigmentosa, bone spicule-like pigmentations were found in the mid periphery of retina (indicated with arrows), and retinal vessels were attenuated (arrowheads).

All three affected individuals (II:4, II:6, and II:7) presented with night blindness that started at an early age. They displayed gradual constriction of their visual field and low visual acuity. Fundus examination revealed typical RP including the bone spicule-like pigment deposits in the mid-peripheral retina and narrowing of the retinal vessels (Figure 2). The unaffected sibling (II:2) did not experience night blindness or any other symptoms of RP. Apart from slightly decreased visual acuity, which is probably age-related, the proband's father (I:1) did not display any ophthalmological symptoms either. The clinical data for all participating individuals are summarized in Table 2.

**Table 2:** Clinical characteristics of participating family members

**Person**	**Current age (y)**	**Gender**	**Age of onset (y)**	**Visual acuity**		**Fundus appearance**
				**OD**	**OS**	
II:4	37	F	8	LP+	LP+	AA, BS, POD
II:6	29	F	9	1/300	1/300	AA, BS, POD
II:7	27	F	11	1/300	2/60	AA, BS
I:1	64	M	NA	20/40	20/25	Normal
II:2	42	M	NA	20/15	20/20	Normal

Clinical characteristics of affected family members carrying two RP1 variants and non-affected family members carrying one RP1 variant or two wild-type alleles. Abbreviations: AA: attenuated arterioles; BS: bone spicules; F: female; LP+: light perception; M: male; NA: not affected; OD: oculus dexter, right eye; OS: oculus sinister, left eye; POD: pale optic disc.

Genome-wide SNP array analysis revealed four homozygous regions larger than 1 Mb that were shared among the three affected siblings (Table 3). Whereas no obvious candidate genes resided in the three largest homozygous regions, the fourth largest homozygous region (1.3 Mb, on chromosome 8q12.1) encompassed *RP1*.

**Table 3:** Overview of homozygous regions shared between the three affected individuals.

**Chr**	**Start position (hg 18)**	**End position (hg 18)**	**Flanking SNP 1**	**Flanking SNP 2**	**Size (Mb)**	**Rank**	**Known arRP gene in the region**	**Mutation (DNA)**	**Mutation (protein)**
16	77.098.649	80.083.138	rs8056971	rs12445748	3	1			
8	50.357.738	52.191.973	rs17664823	rs10090890	2	2			
16	65.737.699	67.289.958	rs11643507	rs1886699	1.5	3			
8	55.432.038	56.765.392	rs7827900	rs7011147	1.3	4	*RP1*	c.1012C>T	p.R338*

Shown are all homozygous regions shared by three affected siblings and larger than 1 Mb. Flanking SNPs and their physical positions correspond to the hg18 NCBI genome assembly. The fourth largest homozygous region harbors *RP1*.

Sequence analysis of *RP1* revealed a novel homozygous substitution (c.1012C>T) in the proximal portion of exon 4, replacing an arginine residue at position 338 with a stop codon (p.R338*). Sequence chromatograms and segregation analysis of the *RP1* mutation are shown in Figure 1. This nonsense alteration was not present in the unaffected sibling, and was identified heterozygously in the unaffected father, who did not show any symptoms of RP. To rule out the possibility that the second allele (inherited from the mother) harbors a deletion of *RP1* responsible for the loss of heterozygosity, we employed copy number analysis of our genome-wide SNP data and did not find any abnormalities in this genomic region. The mutation was not detected in 184 ethnically matched and unrelated Indonesian control subjects.

The *RP1* gene consists of four exons and gives rise to a transcript of approximately 7.1 kb encoding a protein of 2,156 amino acids. The RP1 protein is localized in the region of the connecting cilium and axoneme of photoreceptor sensory cilia [7]. RP1 is a photoreceptor-specific microtubule-associated protein that regulates the length and stability of the photoreceptor axoneme mediated by the two putative doublecortin domains at the N-terminus of RP1 [33]. The doublecortin domain is essential for the correct localization and proper stacking of outer segment discs [34-36]. Moreover, a region of the RP1 protein between codons 486 and 635 bears some homology to the *Drosophila melanogaster* bifocal protein that is required for normal photoreceptor morphogenesis in the fruit fly [7].

**Figure 3:**
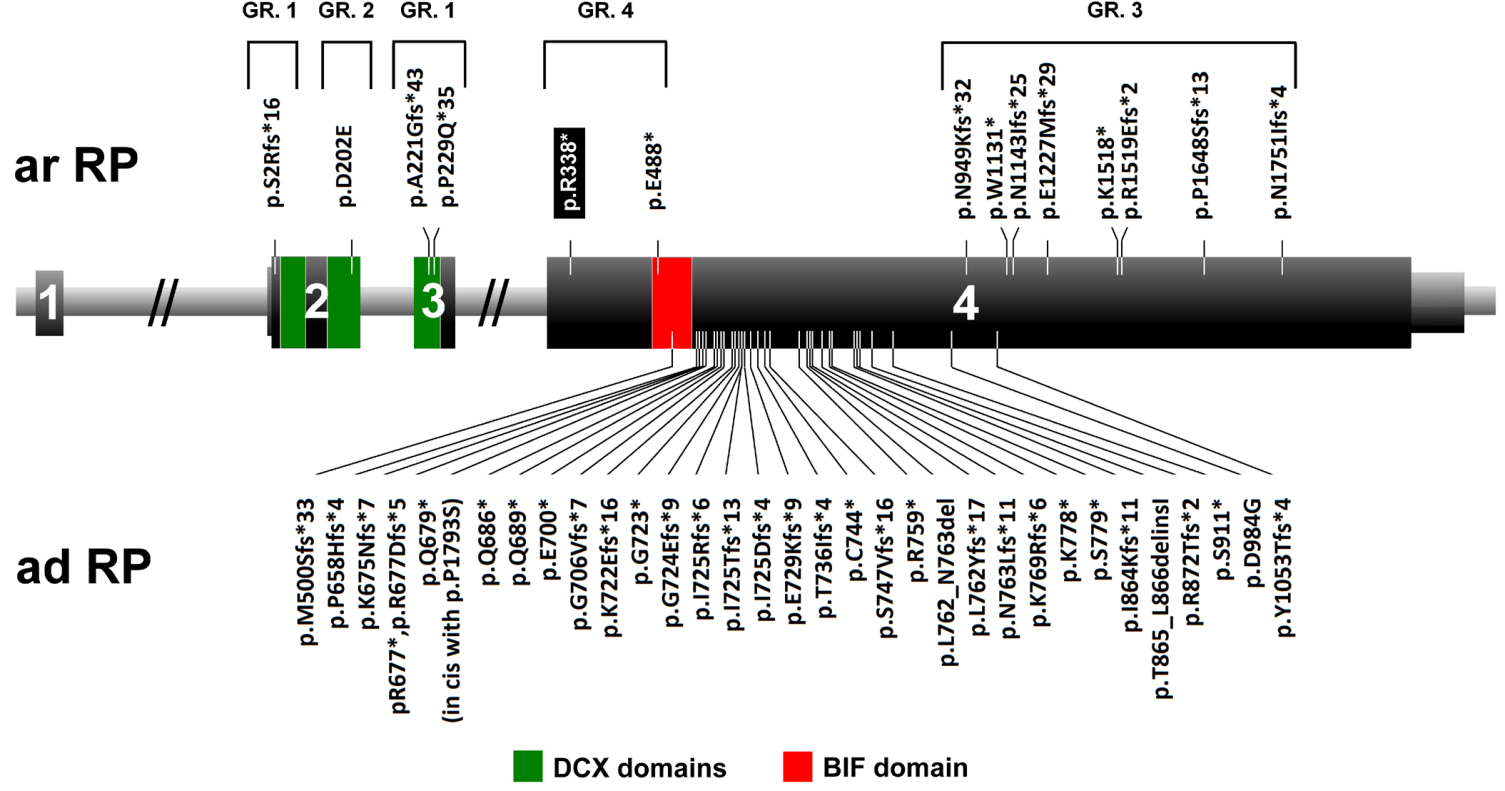
*RP1* gene and identified mutations. Schematic representation of the location of mutations in the *RP1* gene. Below are mutations responsible for dominant retinitis pigmentosa (adRP) whereas mutations above the gene cause autosomal recessive RP (arRP). The mutation identified in this study is indicated with a black rectangle. The portion of the gene that encodes the doublecortin (DCX) domains (amino acids 36–118 and 154–233) is indicated with green, and the *Drosophila melanogaster* bifocal (BIF) domain (amino acids 486−635) is depicted in red. Missense changes, for which the pathogenicity is uncertain, are not included in this scheme. Four groups of arRP-causing mutations are marked with numbers. GR.1=protein-truncating mutations that reside in exon 2 or exon 3 (nonsense-mediated mRNA decay); GR.2=missense mutations; GR.3=protein-truncating mutations near the 3′ end of the gene, preserving residual activity; GR.4=truncating mutations located in the proximal part of exon 4.

The many mutations in exon 4 of *RP1*, which cause autosomal dominant RP, all tend to cluster in a “hot spot” region between amino acid residues 500 and 1053 (Figure 3) [6-24]. Since these mutations are expected to be insensitive to nonsense-mediated decay due to the absence of a downstream intron, they result in the production of truncated proteins [33]. Previously, mutant *RP1* mRNA was found to be present in a human lymphoblast cell line carrying the p.R677* variant, providing evidence that this mRNA indeed escapes the nonsense-mediated mRNA decay process. The resulting truncated proteins lack important functional domains, but are still able to interact with wild-type RP1 and/or microtubule-associated proteins [35]. This hypothesis is supported by a previous study, which showed that in *Rp1-myc* mice that expressed only the 661 N-terminal amino acids of *RP1,* truncated proteins are produced and localize correctly to the axonemes of photoreceptor cells [36]. Due to the retained ability to interact with microtubule-associated proteins and/or wild-type RP1, these mutated proteins are thought to abolish the function of the normal RP1 molecules in a dominant-negative fashion.

For *RP1* mutations that cause recessive RP, the story is more complex. These mutations can be clustered in four different groups (Figure 3). First, protein-truncating mutations that reside in exon 2 or exon 3 are expected to undergo nonsense-mediated decay, and therefore can be considered true loss-of-function alleles [26,28,29]. The second group consists of missense mutations that appear not to cluster [25,28]. These changes cause autosomal recessive RP because the produced proteins retain residual function and therefore are not pathogenic when present in the heterozygous state. The third group includes protein-truncating mutations near the 3′ end of the gene [6,20,26,27]. Most likely, the resulting proteins display only a minor loss of their C-terminal portion, preserving the majority of functional domains, and have residual activity. Therefore, these variants are also pathogenic only when present homozygously. Finally, the most interesting group of mutations encompasses the few nonsense changes located in the proximal part of exon 4 [20,26], and includes the mutation identified in this study. Since the mRNAs deriving from these alleles are expected to escape nonsense-mediated mRNA decay, truncated RP1 proteins are likely to be produced. However, unlike the adRP-causing proteins, the truncated RP1 proteins apparently lack some domains that are crucial for the interactions with wild-type RP1 or microtubule-associated proteins, and therefore no dominant-negative effect emerges. This is supported by the absence of clinical symptoms in the father, who carried the p.R338* mutation heterozygously.

Taken together, the results of our study indicate that early protein-truncating mutations in *RP1*, including p.R338*, cause autosomal recessive RP, supporting the idea that haploinsufficiency is probably not causative for autosomal dominant RP. A better understanding of the pathophysiology of *RP1* mutations may ultimately lead to an improved prognosis, and provide new tools for therapeutic interventions.
